# Selective Targeting of a Novel Vasodilator to the Uterine Vasculature to Treat Impaired Uteroplacental Perfusion in Pregnancy

**DOI:** 10.7150/thno.19678

**Published:** 2017-08-29

**Authors:** Natalie Cureton, Iana Korotkova, Bernadette Baker, Susan Greenwood, Mark Wareing, Venkata R Kotamraju, Tambet Teesalu, Francesco Cellesi, Nicola Tirelli, Erkki Ruoslahti, John D Aplin, Lynda K Harris

**Affiliations:** 1 Maternal and Fetal Health Research Centre, Division of Developmental Biology and Medicine, University of Manchester, Manchester, UK;; 2 Academic Health Science Centre, St Mary's Hospital, Oxford Road, Manchester, M13 9WL, UK;; 3 Cancer Center, Sanford-Burnham Medical Research Institute, 10901 N. Torrey Pines Road, La Jolla, CA 92037, USA and Center for Nanomedicine and Department of Cell, Molecular and Developmental Biology, University of California, Santa Barbara, Santa Barbara, CA 93106-9610, USA;; 4 Laboratory of Cancer Biology, Institute of Biomedicine, Centre of Excellence for Translational Medicine, University of Tartu, Tartu, Estonia;; 5 Dipartimento di Chimica, Materiali ed Ingegneria Chimica "G. Natta". Politecnico di Milano, Via Mancinelli 7, 20131 Milan, Italy;; 6 Fondazione CEN - European Centre for Nanomedicine, Piazza Leonardo da Vinci 32, 20133 Milan, Italy;; 7 Division of Pharmacy and Optometry, University of Manchester, Stopford Building, Oxford Road, Manchester, M13 9PT, UK.

**Keywords:** pregnancy, placenta, nitric oxide, endothelium, vascular biology, drug targeting.

## Abstract

Fetal growth restriction (FGR) in pregnancy is commonly caused by impaired uteroplacental blood flow. Vasodilators enhance uteroplacental perfusion and fetal growth in humans and animal models; however, detrimental maternal and fetal side effects have been reported. We hypothesised that targeted uteroplacental delivery of a vasodilator would enhance drug efficacy and reduce the risks associated with drug administration in pregnancy. Phage screening identified novel peptides that selectively accumulated in the uteroplacental vasculature of pregnant mice. Following intravenous injection, the synthetic peptide CNKGLRNK selectively bound to the endothelium of the uterine spiral arteries and placental labyrinth *in vivo*; CNKGLRNK-decorated liposomes also selectively bound to these regions. The nitric oxide donor 2-[[4-[(nitrooxy)methyl]benzoyl]thio]-benzoic acid methyl ester (SE175) induced significant relaxation of mouse uterine arteries and human placental arteries *in vitro*; thus, SE175 was encapsulated into these targeted liposomes and administered to healthy pregnant C57BL/6J mice or endothelial nitric oxide synthase knockout (eNOS^-/-^) mice, which exhibit impaired uteroplacental blood flow and FGR. Liposomes containing SE175 (0.44mg/kg) or PBS were administered on embryonic (E) days 11.5, 13.5, 15.5 and 17.5; fetal and placental weights were recorded at term and compared to mice injected with free PBS or SE175. Targeted uteroplacental delivery of SE175 had no effect on fetal weight in C57BL/6J mice, but significantly increased fetal weight and mean spiral artery diameter, and decreased placental weight, indicative of improved placental efficiency, in eNOS^-/-^ mice; free SE175 had no effect on fetal weight or spiral artery diameter. Targeted, but not free SE175 also significantly reduced placental expression of 4-hydroxynonenal, cyclooxygenase-1 and cyclooxygenase-2, indicating a reduction in placental oxidative stress. These data suggest that exploiting vascular targeting peptides to selectively deliver SE175 to the uteroplacental vasculature may represent a novel treatment for FGR resulting from impaired uteroplacental perfusion.

## Introduction

More than five percent of pregnancies are complicated by fetal growth restriction (FGR), where the developing fetus does not reach its full growth potential, resulting in significant maternal and fetal morbidity and/or mortality 1,2. There is also evidence that being born small or premature can program obesity and increase the risk of adulthood diseases such as cardiovascular disease and type 2 diabetes 3. Current management strategies are limited to fetal monitoring, and induction of labour when suboptimal growth threatens fetal survival 4. However, pre-term delivery can exacerbate some of the adverse effects associated with growth restriction 5. Despite these short and long-term health implications, development of therapeutics for use in pregnancy is hindered by the risk of systemic side effects and lack of interest from the pharmaceutical industry 6,7.

Peptide-mediated targeting of payloads to individual vascular beds has enabled selective delivery of therapeutics to specific organs and tissues 8,9, offering enhanced efficacy and importantly, reducing off-target, systemic side effects 10. Much of the current research in this area has focussed on delivery of chemotherapeutics to tumours, which has been achieved by targeting specific receptors on the tumour vasculature 11, 12. A peptide that homes to atherosclerotic plaques has also been identified, which triggers apoptosis of plaque macrophages and reduces plaque hypoxia 13,14. The villous placenta and surrounding uterine vasculature express a number of cell-surface epitopes common to those observed in the vasculature in solid tumours 15. Indeed, the placenta has been likened to a solid tumour, exhibiting high rates of proliferation in the first trimester, giving rise to a subset of invasive cells and modulating the maternal immune response. These observations, along with our finding that some peptides which home to tumours can also be exploited for placental targeting 16, led us to seek novel sequences that home to the uteroplacental vasculature.

Abnormal vascular anatomy 17 and impaired myometrial and chorionic plate vascular tone regulation are present in some cases of FGR in humans 18-22. Reduced uterine and umbilical blood flow alters the diffusion gradient for transfer of small lipophilic solutes such as O_2_ and CO_2_ across the placenta 23, thus restricting fetal growth. Moreover, impaired uteroplacental blood flow contributes to FGR in both human and mouse pregnancies 24,25, suggesting that enhancement of flow may provide a means to improve fetal growth *in utero*. Indeed, administration of the vasodilator sildenafil citrate (Viagra™) to pregnant women presenting with FGR improved uteroplacental perfusion, as identified by Doppler ultrasound 26. Sildenafil citrate also significantly increased fetal weight in the two mouse models of FGR 27-29; however, maternal administration of sildenafil citrate caused impaired vasorelaxation of fetal abdominal aortas, led to female offspring exhibiting impaired glucose tolerance, and caused the offspring to have elevated blood pressure as adults 27,30,31. These studies demonstrate the potential side effects of systemic maternal delivery of therapeutics in pregnancy and lend support to the use of targeted drug delivery.

In this study, we have used phage screening to identify novel peptide sequences that selectively bind to the uteroplacental vasculature and created liposomes bearing one such sequence for targeted drug delivery. We have also assessed the ability of a novel vasodilator, 2-[[4-[(nitrooxy)methyl]benzoyl]thio]-benzoic acid methyl ester (SE175), to relax uterine arteries from pregnant mice and enhance fetal growth *in vivo*. SE175, a nitroxyacylated thiosalicylate, contains a nitric oxide (NO)-donating nitrate group attached to a NO-liberating thiosalicylate 55; this class of compound was created in an effort to achieve spontaneous release of NO from nitrate groups, based on the evidence that a free thiol (SH) is able to liberate NO from nitrate groups 72. Esterase breakdown of SE175 allows the thiosalicylate group to self-liberate NO from the nitrate group, thus SE175 should not cause the tolerance that has been observed following repeated administration of traditional nitrates.

We evaluated the effects of SE175 in pregnant wild-type C57BL/6J mice and in endothelial nitric oxide synthase (eNOS^-/-^) knockout mice, which exhibit pre-pregnancy hypertension and a compromised adaptive cardiovascular response to pregnancy: uterine artery remodeling is impaired 32,33 and uteroplacental perfusion is reduced 34. Offspring are on average 10% lighter than in C57BL/6J mice indicating FGR 35. No gross structural abnormalities have been reported in eNOS^-/-^ mice but placental nutrient transport is reduced, and placentas exhibit evidence of oxidative stress and uteroplacental hypoxia 36. Oxidative stress in the mouse placenta leads to the induction of both cyclooxygenase-1 (COX-1) and cyclooxygenase-2 (COX-2), leading to prostaglandin synthesis and elevated rates of apoptosis 37. Here we demonstrate that targeted delivery of SE175 to the uteroplacental vasculature of eNOS^-/-^ mice enhances fetal growth and reduces placental oxidative stress.

## Methods

### Animal procedures

BALB/c mice (Charles River, USA), C57BL/6J mice (UK) and eNOS mice (UK) were housed and all procedures were performed according to procedures approved by the Animal Research Committees at University of California, Santa Barbara, or in accordance with the UK Animals (Scientific Procedures) Act 1986 at The University of Manchester. Animals had free access to food and water and were maintained on a 12:12 h light-dark cycle at 21-23°C. After mating, the presence of a copulation plug was denoted as embryonic* day 0.5 (E0.5)* of pregnancy.

### Phage display

The T7-select phage display system (EMD Biosciences, USA) was used to construct peptide libraries. Individual phages were cloned according the manufacturer's instructions, as previously described 38,39. Amplified phages were purified from bacterial lysate by precipitation with PEG-8000 (Sigma Aldrich, MO, USA), caesium chloride gradient ultracentrifugation and dialysis. The identity of the displayed peptides was determined by sequencing the DNA encoding the insert-containing region at the C terminus of the T7 major coat protein gp10 (Eton Bioscience, CA, USA).

### Phage screening

Mouse placentas (E13.5 - E14.5) were harvested, washed in PBS and mechanically dissociated using a Medimachine system (BD Biosciences). The resulting tissue aggregates were incubated with the T7 phage library (10^10^ pfu/mg tissue) in 10 ml DMEM culture medium containing 1% (w/v) BSA (Life Technologies, NY, USA) for 1 h at 4°C on a rotating wheel, then pelleted by centrifugation (400*g*; 5 min). Tissue was washed by centrifugation (400 *g*; 5 X 5 min) in fresh culture medium and lysed in LB bacterial growth medium containing 1% Nonidet P-40 (Sigma Aldrich, MO, USA). Bound phage was quantified by titration and amplified as previously described 38,39. The *ex vivo* selection process was repeated three times, then the resulting phage pool was injected into the tail vein of a pregnant mouse and allowed to circulate for 30 min. To remove unbound phage, mice were subjected to terminal cardiac perfusion with 30 ml of PBS. The uterus and placentas were harvested and homogenized in LB bacterial growth medium containing 1% (v/v) Nonidet P-40, and bound phage were titered, amplified and purified. Four rounds of *in vivo* screening were performed in total, using pregnant mice between E13.5 and E15.5. Ninety-six individual phage clones from each of the second, third and fourth rounds of screening were subjected to DNA sequencing to determine the identity of their surface peptides.

### Peptide synthesis

Peptides were synthesized at The University of California, Santa Barbara using Fmoc chemistry in a solid-phase synthesizer, were purified by HPLC, and sequences and structures confirmed by mass spectrometry as previously described 38. Alternatively, they were purchased from Insight Biotechnology, UK.

### Peptide targeting

Individual synthetic peptides (200 µg) labeled with 5(6)-carboxyfluorescein (FAM) were injected into the tail vein of pregnant mice (E11.5-E17.5) and allowed to circulate for 3 h. Following terminal cardiac perfusion with PBS to remove unbound peptide, maternal and fetal tissues were collected for analysis. Organs were snap frozen, or fixed in paraformaldehyde (4% (w/v) in PBS; overnight) and cryoprotected in sucrose solution (30% (w/v) in PBS; 24 h), embedded in OCT (Sakura) and stored at -80 ºC. Tissue sections (8 μm) were fixed in ice-cold methanol (15 min), washed in PBS (2 X 5 min), mounted in Vectashield medium containing DAPI (4′,6-diamidino-2-phenylindole; Vector Laboratories, Burlingame, USA) and examined on an Olympus Fluoview 500 confocal microscope (Olympus America) or a Zeiss AxioObserver fluorescence microscope (Zeiss, UK). Images were captured at the same exposure so that comparisons of fluorescence intensity could be made between samples.

### Liposome synthesis

Targeted liposomes were prepared by dissolving 1,2-distearoyl-*sn*-glycero-3-phosphocholine (DSPC; 65 μmol/L), cholesterol (30 μmol/L), 1,2-distearoyl-*sn*-glycero-3-phosphoethanolamine-N-[amino(polyethylene glycol)] (DSPE-PEG; 4.5 μmol/L) and DSPE-PEG-maleimide (0.5 μmol/L; Avanti Polar Lipids) in methanol:chloroform (9:1 ratio). Solvent was removed by rotary evaporation to produce a thin lipid film, which was rehydrated with distilled water (2 mL). The resulting suspension was heated to 62^o^C for 3 h to produce large multilamellar vesicles and extruded eleven times using a 1 mL Mini-Extruder (Avanti Polar Lipids) through a 0.1 μm, 19 mm polycarbonate membrane, surrounded by two 10 mm filter supports in order to produce a unilamellar liposome suspension. Fluorescent targeting peptides (0.27 μmol/L) bearing a cysteine residue on the N-terminus were added to 1 mL of extruded liposome suspension and allowed to covalently couple with maleimide groups overnight at room temperature via a Michael type addition reaction. Unreacted peptide was removed by dialysis against PBS (8 X 1 litres; 24 h; Slide-A-Lyzer Dialysis Cassettes, MWCO 3.5 kDa) and the suspension stored at 4^o^C until use. The size (hydrodynamic diameter) distribution (SD) and polydispersity index (PDI) were measured by dynamic light scattering (DLS), and the zeta potential (ZP) was measured by laser Doppler micro-electrophoresis (LDE) (25^o^C; scattering angle of 173^o^ (Zetasizer Nano ZS, Model ZEN3600, fitted with a 632 nm laser, Malvern Instruments Ltd., UK).

To encapsulate SE175, the lipid film was rehydrated with SE175 (2 mL, 320 µmol/L stock) and the resultant suspension was heated at 62^o^C for 3 h and extruded, as described above. Fluorescent targeting peptides (0.27 μmol/L) were conjugated to maleimide groups on the liposomal surface via a Michael-type addition reaction. Unreacted peptide was removed by dialysis against PBS and the suspension was stored at 4^o^C.

Non-targeted liposomes were prepared by dissolving DPSC (65 μmol/L), cholesterol (30 μmol/L), DSPE-PEG (5 μmol/L) in methanol:chloroform (9:1 ratio), followed by the addition of 2.7 μmol/L of fluorescently labelled lipid NBD-DSPE (5 0μL, 0.1 mg/mL stock). As described above, the solvent was removed by rotary evaporation, the lipid film was rehydrated with PBS (2 mL) and the resultant suspension was heated at 62^o^C for 3 h. The suspension was then extruded using a 1 mL Mini-Extruder through a 0.1 μm, 19 mm polycarbonate membrane, free drug was removed by dialysis against PBS and the suspension was stored at 4^o^C.

### Validation of liposome targeting *in vivo*

Liposomes (100 µL) decorated with rhodamine-labelled CNKGLRNK peptides and NBD-labelled fluorescent lipids were injected into the tail vein of pregnant mice (E13.5-E15.5) and allowed to circulate for up to 48h. Following terminal cardiac perfusion with PBS to remove unbound liposomes, maternal and fetal tissues were collected for analysis. Organs were fixed in neutral buffered formalin (4% (v/v); overnight), embedded in OCT (RA Lamb, UK) and stored at -80 ºC. Tissues sections (8 μm) were fixed in PFA (15 min), washed in PBS (2 X 5 min), mounted in Vectashield mounting medium containing DAPI and examined using a Zeiss AxioObserver fluorescence microscope (Zeiss, UK). Images were captured at the same exposure so that comparisons of fluorescence intensity could be made between samples.

### Human tissue

First trimester placentas were collected following surgical or medical termination of pregnancy. Term placentas (37-42 weeks gestation) were obtained from normal pregnancies within 30 min of vaginal or Caesarean delivery. The study was performed in accordance with North West Local Research Ethical Committee approvals (08/H1010/28; 08/H1010/55) and written informed consent was obtained from all patients. Villous tissue was randomly sampled, washed in serum-free culture medium and dissected into 3 mm^3^ explants under sterile conditions. Explants were cultured a 1:1 ratio of DMEM and Ham's F12 media (Lonza Biosciences, UK) containing glutamine (2 mmol/L), penicillin (100 IU ml^-1^), streptomycin (100 µg ml^-1^) and 10% (v/v) fetal bovine serum (Life Technologies), in 24 well culture plates pre-coated with agarose (1% (w/v); Sigma Aldrich). Explants were maintained in 20% O_2_, 5% CO_2_ at 37 °C for up to 7 days.

### Peptide binding to human placental explants

Rhodamine-labelled peptides (20 µmol/L) were incubated with human first trimester or term placental explants at 37ºC for up to 3 h in culture media. Tissue was washed in PBS, fixed in neutral buffered formalin overnight (4% (v/v); pH 7.4), frozen and cryosectioned. Tissues sections (8 μm) were fixed in PFA (15 min), washed in PBS (2 X 5 min), mounted in Vectashield mounting medium containing DAPI and assessed using a Zeiss AxioObserver fluorescence microscope.

### Liposome binding to human placental explants

Peptide-decorated liposomes (100 µL) were cultured with term placental explants (37^o^C, 95% air, 5% carbon dioxide) for up to 48 h. Tissue was washed in PBS, fixed in neutral buffered formalin overnight (4% (v/v); pH 7.4), frozen and cryosectioned. Tissue sections (8 μm) were fixed in PFA (15 min) and washed in PBS (2 X 5 min). Sections were mounted in Vectashield containing DAPI and visualised using a Zeiss AxioObserver fluorescence microscope.

### Wire myography

Pregnant female mice were euthanized by cervical dislocation at E18.5. A surgical laparotomy was performed on the dam to expose the uterine horn. The horn was pinned out as previously described 36 and main loop uterine artery carefully excised using a dissection microscope (model S6E, Leica, UK). The dissected artery was trimmed into approximately 40 µm length sections and kept in cold physiological salt solution prior to mounting on the wire myograph. Dissected arterial sections were mounted by inserting two 40 µm steel wires into the lumen, which were secured in the jaws of a Multi Myograph System 610M (Danish Myo Technology A/S, Denmark) as previously described 40. Arterial section diameter was measured using a calibrated eyepiece graticule and equilibrated to physiological conditions in 6 mL PSS, warmed to 37^o^C and gassed with 20% O_2_ / 5% CO_2_ / balanced with N_2_ (BOC Gases, Worsley, UK). Vessels were normalised to ensure that arterial sections of different diameters had the same resting tension, as previously described 41, and were left to equilibrate for 20 minutes.

Chorionic plate biopsies taken from human term placentas were placed into ice-cold PSS. Small chorionic plate arterial branches originating from the main umbilical arteries were identified, dissected and trimmed into approximately 40 µm length sections. Vessels were mounted on a wire myograph and normalised as described above.

Human chorionic plate and mouse uterine arteries were exposed to a high potassium salt solution for 5 min to determine maximal smooth muscle contraction, before being washed twice with PSS. Arterial sections were left to re-equilibrate for 20 min, before being exposed to phenylephrine (PE, 10^-5^ mol/L) for 10 min to determine maximal contraction. After 10 min, arterial sections were exposed to acetylcholine (ACh, 10^-5^ mol/L) to determine maximal relaxation, before being washed twice with PSS and left to equilibrate for 20 min. PE exposure was then repeated and relaxation to SE175 determined, following the application of incremental doses (10^-9^ - 10^-5^ M, 2 min intervals). Equivalent concentrations of DMSO (vehicle control) and no treatment (time control) were assessed in parallel. When targeted liposomes containing SE175-were applied to arterial sections, the extent of relaxation to a single dose (100 µl) of SE175 was determined. Relaxation data were expressed as percentage relaxation of maximum pre-constriction to PE. Myodata software (version 2.02; Myonic Software, National Instruments Corporation, USA) was used to visualise and interpret data.

### Measurement of system A activity

After 6 days in culture, term placental explants were treated with SE175 (10 µM), SNAP (10 µM) or DMSO (0.6 %; vehicle control) and system A activity was measured on day 7. Explants were incubated for 30, 60 or 90 min in either Na^+^-containing or Na^+^-free Tyrode's buffer (NaCl replaced by an equimolar concentration of choline chloride) containing 0.5 μCi/ml ^14^C-MeAIB (∼8.5 μM) at 37 °C. The explants were then washed in ice-cold Tyrode's buffer and lysed in dH_2_O for 16-18 h at room temperature (RT). The radioactive content of the water lysate was measured using a β-counter. Na^+^-dependent system A activity was calculated by subtracting uptake in Na^+^-free Tyrode's from that in Na^+^-containing Tyrode's, with correction for fragment protein content, measured using Bio-Rad protein assay (Bio-Rad Laboratories Ltd, Hertfordshire, UK).

### Liposome treatment study

Time-mated, pregnant C57 and eNOS^-/-^ mice were intravenously injected with 100 µL of either PBS, CNKGLRNK-decorated liposomes containing PBS, free SE175 (320 µmol/L, approximately 0.44 mg/kg) or CNKGLRNK-decorated liposomes containing SE175 (320 µmol/L; approximately 0.44 mg/kg) on E11.5, E13.5, E15.5 and E17.5. Mice were sacrificed by cervical dislocation at E18.5 and the following measurements taken: litter size, number of resorptions, fetal weight, placental weight, maternal kidney weight, maternal spleen weight and maternal heart weight. Organs were fixed in neutral buffered formalin (4% (v/v), pH 7.4; overnight) for paraffin embedding. The weight and behaviour of treated dams were continuously monitored to detect signs of toxicity or distress. Gross morphological analysis of fetuses and maternal organs was undertaken to detect physical deformities or abnormalities associated with treatment.

### Immunostaining

Tissue sections (5 µm) were deparaffinised in Histoclear and alcohol and then rehydrated in dH_2_O. Slides were microwaved in antigen retrieval buffer (sodium citrate buffer (0.01 mol/L), containing 0.05% (v/v) Tween 20 (pH 6.0), 20 min), followed by cooling for 10 min. After cooling, tissue sections were incubated with hydrogen peroxidase (3% (w/v), 10 min) to block endogenous peroxide activity, washed 3 times in Tris-buffered saline (TBS) and incubated with bovine serum albumin (BSA; 5% (w/v) in TBS, 30 min) to block non-specific binding. Tissue sections were then incubated with primary antibodies (Table 1) or control IgG (matched concentration; Sigma Aldrich) overnight at 4^o^C. Tissue sections were washed 3 times (TBS; 5 min) and incubated at room temperature with a swine anti-rabbit or goat anti-mouse secondary antibody (1:200; DakoCytomation A/S) for 30 min. Tissue sections were then washed 3 times (TBS; 5 min) and incubated at room temperature with avidin peroxidase (5 µg/mL in TBS, 30 min; Sigma Aldrich). Tissue sections were then washed with dH_2_O, incubated for 2-10 min with diaminobenzidine (DAB; 0.05% (w/v); Sigma Aldrich) and urea hydrogen peroxide (0.015% (v/v); Sigma Aldrich, UK) and washed again with dH_2_O. Tissue sections were then counterstained with filtered Harris's hematoxylin, dehydrated in alcohol and Histoclear and mounted in DPX mountant (Sigma Aldrich, UK). All sections were stained in the same run to allow direct comparison between groups.

For quantitative analysis of immunostaining, a minimum of three treated mice per group were selected at random and three placentas per mouse were immunostained in the same run for direct comparison of images. Nine images were taken per placenta using the same exposure settings, on the Olympus B641 Light Microscope using Image-Pro Plus 7 software, equating to twenty-seven images per mouse (taken as n = 1). Images were then analysed using TissueGnostics HistoQuest® Analysis Software. Hematoxylin (nuclear stain) total area and DAB (antibody stain) total area were determined and a ratio of DAB area to hematoxylin area calculated to control for tissue area. The mean luminal diameter of individual spiral arteries was also quantified using HistoQuest® software. For human tissue, six random images were taken of each explant using the same exposure settings and analysed using the HistoQuest software. The percent of proliferating and apoptotic cells was calculated by dividing the total area of DAB-positive nuclei (Ki67 or M30 positive cells) by the total area of haematoxylin-positive nuclei and multiplying by 100.

### Statistical analysis

All data were analysed using GraphPad Prism software (Version 7). Myography data were analysed using two-way analysis of variance with Bonferroni *post hoc* test for comparisons between groups. System A activity was analysed using linear regression to assess differences in the rate of Na^+^-dependent ^14^C-MeAIB uptake between treatments. Unless otherwise stated, all other data were analysed using one-way ANOVA or a Kruskal Wallis test with Dunnett *post hoc* test for comparisons between groups. Frequency distribution curves were generated by performing non-linear regression on fetal / placental weight histograms. Unless otherwise stated, data are shown as mean 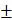 SEM and significance was taken as *P* < 0.05.

## Results

### The placental-homing peptide NKGLRNK selectively binds to the uteroplacental vasculature

**Figure 1:**
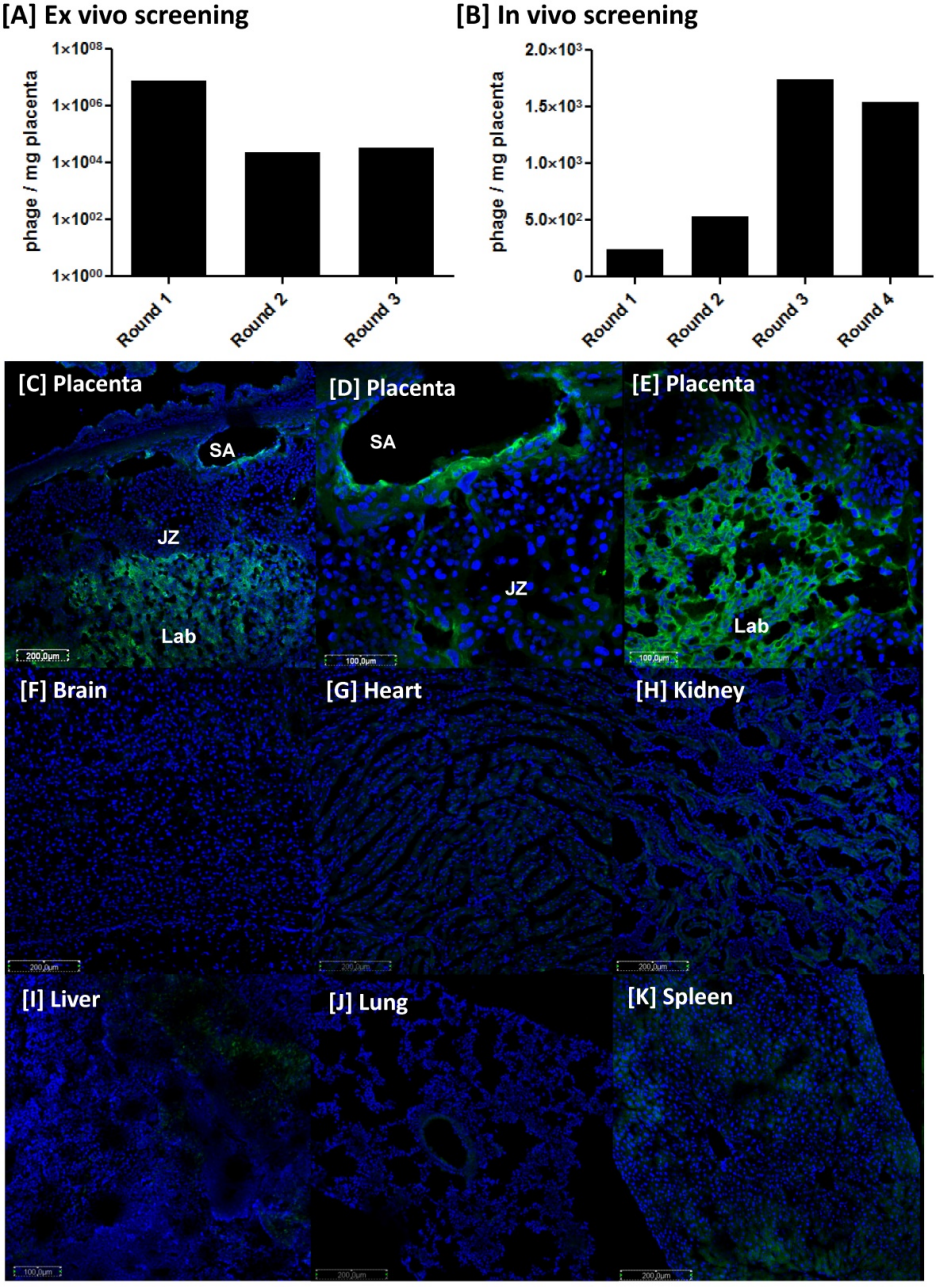
** Identification of the placental-homing peptide NKGLRNK [A]** Mouse placental tissue was incubated with a T7 bacteriophage library and bound phages were titered, amplified and purified. Phage titer (pfu/mg tissue) was measured using a plaque-forming assay (n=3). **[B]** Following 3 rounds of *ex vivo* selection, the resulting phage pool was injected into the tail vein of a pregnant mouse. Placentas were harvested and bound phage pools were titered, amplified and purified. Four rounds of *in vivo* screening were performed in total; phage titer is expressed as pfu/mg tissue (n = 3). **[C-E]** NKGLRNK labelled with 5(6)-carboxyfluorescein (FAM; 200 μg) was injected into the tail vein of pregnant mice (n = 5; E10.5 - E17.5). Following cardiac perfusion to remove unbound peptide, placentas were harvested and assessed by fluorescence microscopy. Representative images are shown. FAM-labelled peptides, green; DAPI (nuclei), blue. JZ = junctional zone; Lab = labyrinth; SA = spiral artery.

To isolate placental homing phages, mouse placental tissue (E13.5-14.5) was incubated with a T7 bacteriophage library containing about 10^8^ individual clones, in which each clone displays multiple copies of a unique, random 7-mer peptide on its surface. Each sequence is represented ~100 times, thus the starting library contained approximately 10^10^ phage. During the three rounds of *ex vivo* phage selection, the phage library collapsed by three orders of magnitude (10^7^ to 10^4^; Figure 1A), as previously reported 39; however, subsequent rounds of *in vivo* screening led to phage enrichment (Figure 1B). Ninety-six phage clones from the second, third and fourth rounds of *in vivo* screening were sequenced and the surface peptide NKGLRNK (NKG) was the most highly represented. To confirm that this sequence selectively targets the mouse placenta *in vivo*, synthetic peptide labelled with 5(6)-carboxyfluorescein (FAM) was injected intravenously into pregnant mice. FAM-NKG bound to the intima of decidual spiral arteries (SA) and the vasculature of the placental labyrinth (Lab), but not to the junctional zone (JZ) between E10.5 and E17.5 (Figure 1C-E). Analysis of maternal tissues confirmed that FAM-NKG did not accumulate in the vascular bed of any other major organ, although small, discrete areas of fluorescence were sometimes observed in the kidney and spleen (Figure 1F-K).

### Homing peptide-decorated liposomes preferentially home to the uteroplacental vasculature

**Figure 2:**
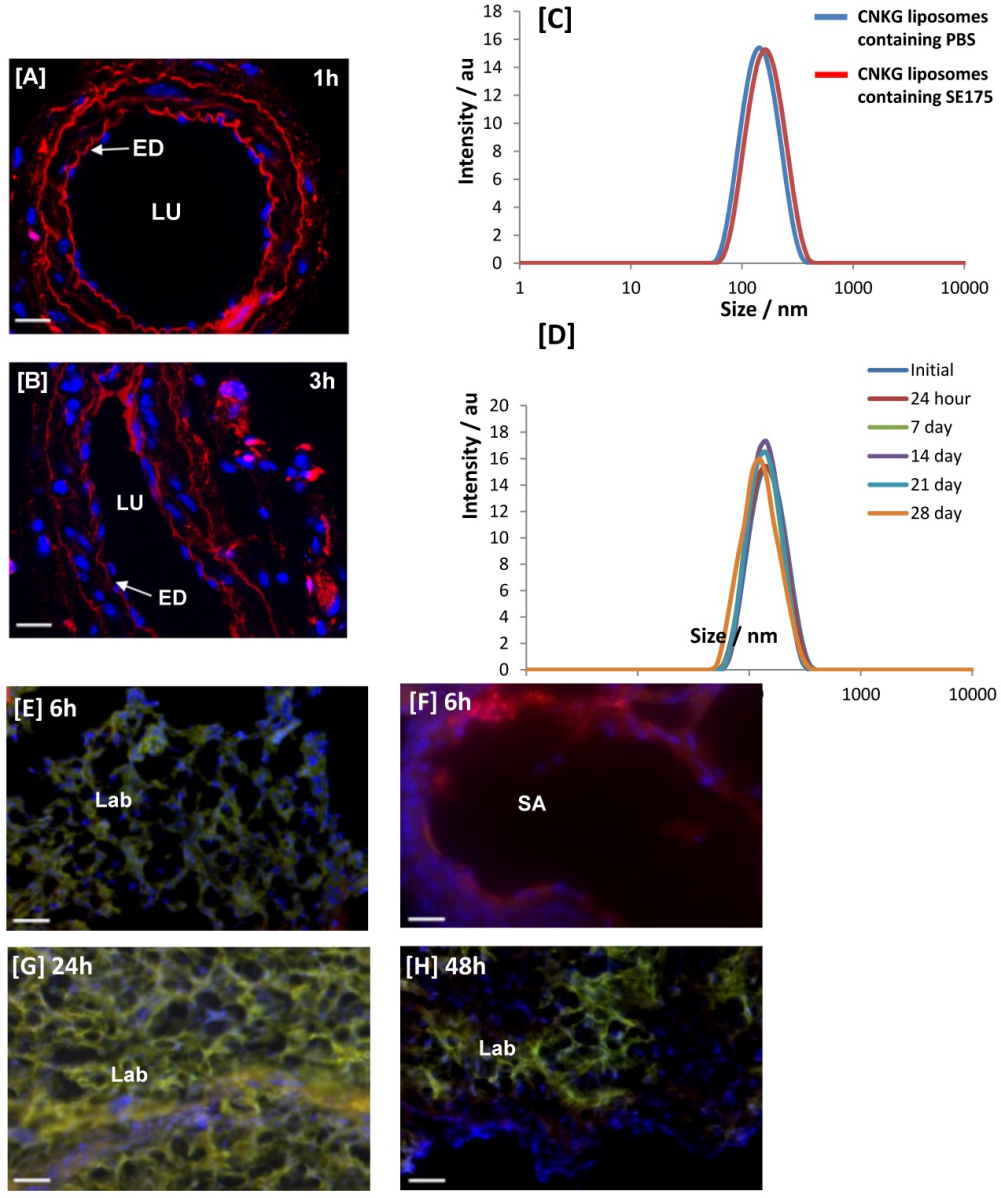
** CNKGLRNK binding to uteroplacental tissue *in vitro* and* in vivo* [A, B]** Segments of mouse uterine artery were incubated with Rh-CNKGLRNK (0.27 µmol/L; red) for up to 3 h. Peptide binding and uptake was assessed by fluorescence microscopy (n = 3). Representative images are shown. Rh-labelled peptides, red; DAPI (nuclei), blue. ED = endothelium; LU = lumen. Scale bar = 50 µm. **[C]** Size distribution of freshly prepared Rh-CNKGLRNK-decorated liposomes containing either PBS (blue) or SE175 (red). A representative plot from n = 3 liposome preparations is shown. **[D]** Size distribution of Rh-CNKGLRNK-decorated liposomes containing PBS over a 28 day period (n=3). Representative plots from one preparation are shown. **[E - H]** Placentas from pregnant C57BL/6J mice collected at E18.5, following tail vein injection of Rh-CNKGLRNK (red)-decorated liposomes composed of NBD-labelled lipids (green), 6h, 24h or 48h prior to tissue harvest. Cardiac perfusion was performed to remove unbound peptide; organs were removed and assessed by fluorescence microscopy. Yellow, co-localisation of peptide (red) and lipid (green) fluorescence. Blue, DAPI (nuclei). SA, spiral artery; LB, labyrinth. Scale bars = 50 µm. n = 3 placentas from n = 3 mice per treatment group were examined. Representative images are shown.

To conjugate the homing peptide to the surface of liposomes it was necessary to add a cysteine residue to the N-terminus: CNKGLRNK (CNKG). To ensure that tissue binding was not affected, excised mouse uterine arteries were incubated with rhodamine (Rh)-labelled peptide (20 µmol/L) for up to 3 h. Rh-CNKG bound to, and accumulated within, the vascular endothelium and underlying smooth muscle layers (Figure 2A, B), as observed *in vivo*. Rh-CNKG-coupled liposomes containing PBS or SE175 were synthesised and characterised; PBS-loaded liposomes had a mean diameter of 159.8 ± 1 nm, a mean zeta potential of -0.6 ± 0.4 mV and a polydispersity index of 0.065. SE175-loaded liposomes had a diameter of 164.2 ± 1 nm, a zeta potential of -1.0 ± 0.6 mV and a polydispersity index of 0.049 (Figure 2C, D). The liposomes demonstrated excellent stability over 28 days, with mean particle size and polydispersity index remaining constant during this time.

To validate liposome targeting *in vivo*, Rh-CNKG-coupled liposomes prepared using fluorescent NBD lipids (green) were injected intravenously into pregnant mice at E15.5 and allowed to circulate for 6 h, 24 h or 48 h. Colocalisation of peptide and lipid fluorescence was observed in the placental labyrinth and the decidual spiral arteries (Figure 2E-H), with a pattern of localisation similar to free NKG peptide. Fluorescence was not observed in the maternal heart, lungs, brain or in any fetal tissues; however a transient signal was seen in the maternal liver, kidney and spleen (Supplementary Figure 1), in line with previous reports of non-specific uptake of nanoparticles by the clearance organs 42.

### The nitroxyacylated thiosalicylate SE175 induces dilation of uteroplacental arteries in mice and humans

**Figure 3:**
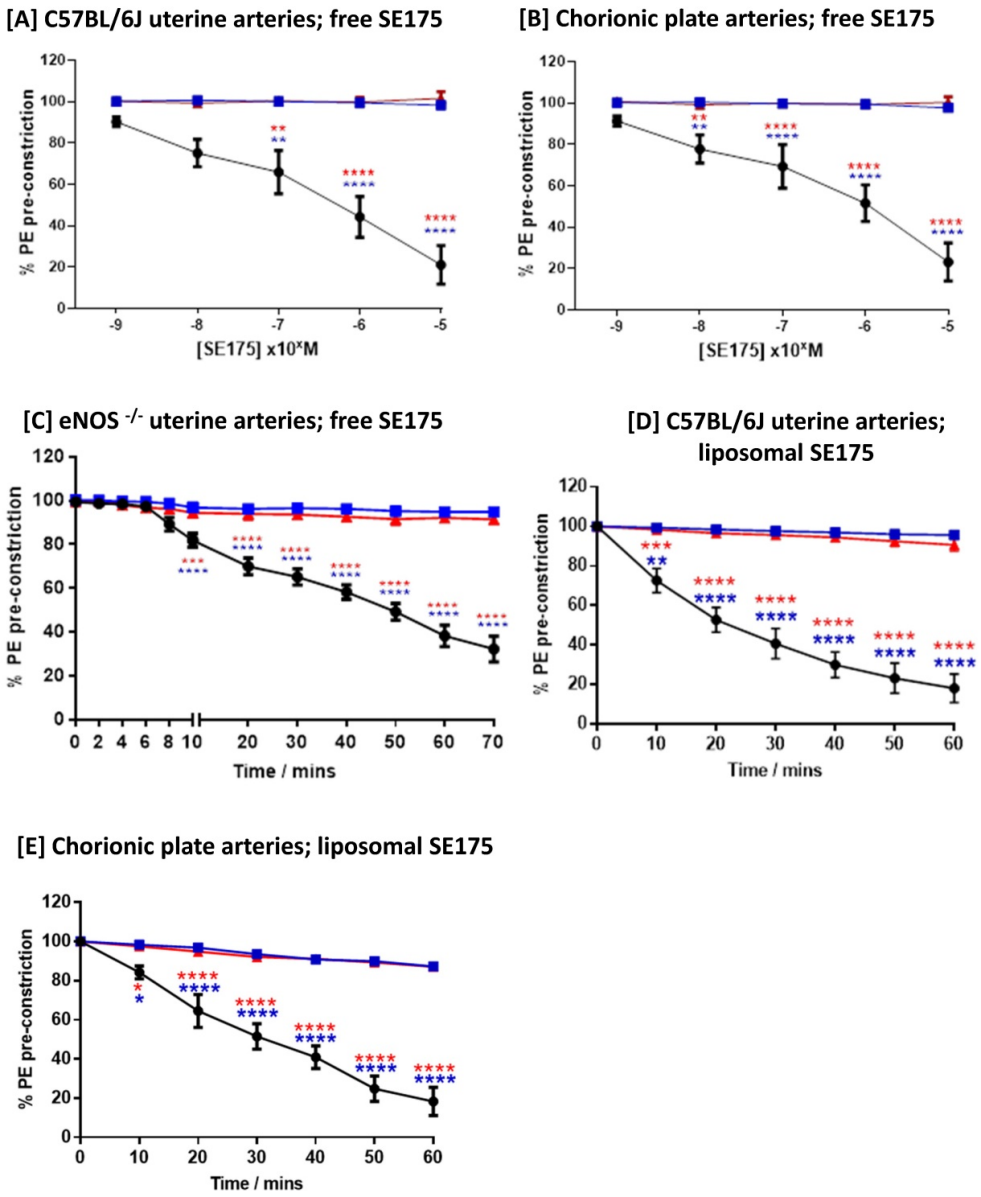
** Human and mouse vessels relax in response to SE175 *ex vivo* [A, D]** C57BL/6J mouse uterine arteries, **[B, E]** human term placental chorionic plate arteries, and **[C]** eNOS^-/-^ mouse uterine arteries were collected, the arteries dissected and mounted onto a wire myograph. Vessels were pre-constricted with phenylephrine and vessel relaxation to **[A - C]** free or **[D, E]** liposomally-encapsulated SE175 was recorded. **[A, B]** Dose-dependent relaxation; **[C-E]** time-dependent relaxation to 10^-5^ M SE175. SE175 (black circles, n = 6), DMSO (blue squares, n = 6), time control (no treatment; red triangles, n = 6). Mean ± SEM; error bars not visible if smaller than symbol. Dose response curves were compared using two-way ANOVA with Bonferroni's post hoc test. **P<0.001, ***P<0.0008, ****P<0.0001.

Free SE175 was applied in increasing concentrations to freshly isolated uterine arteries from C57BL/6J and eNOS^-/-^ mice, or human chorionic plate arteries from healthy term placentas, pre-constricted with phenylephrine. SE175 induced a significant, dose-dependent relaxation of C57BL/6J and chorionic plate vessels (Figure 3A, B), compared to untreated arteries and arteries treated with vehicle control; at a concentration of 10^-5^ M, SE175 induced a significant time-dependent relaxation of eNOS^-/-^ uterine arteries (Figure 3C). Similarly, when encapsulated in CNKG-decorated liposomes, 10^-5^ M SE175 induced a significant, time-dependent relaxation of C57BL/6J and chorionic plate arteries, compared to untreated vessels and those treated with the vehicle control (Figure 3D, E).

### Targeted delivery of SE175 increases fetal weight, placental efficiency and decidual spiral artery diameter in eNOS^-/-^ mice

**Figure 4:**
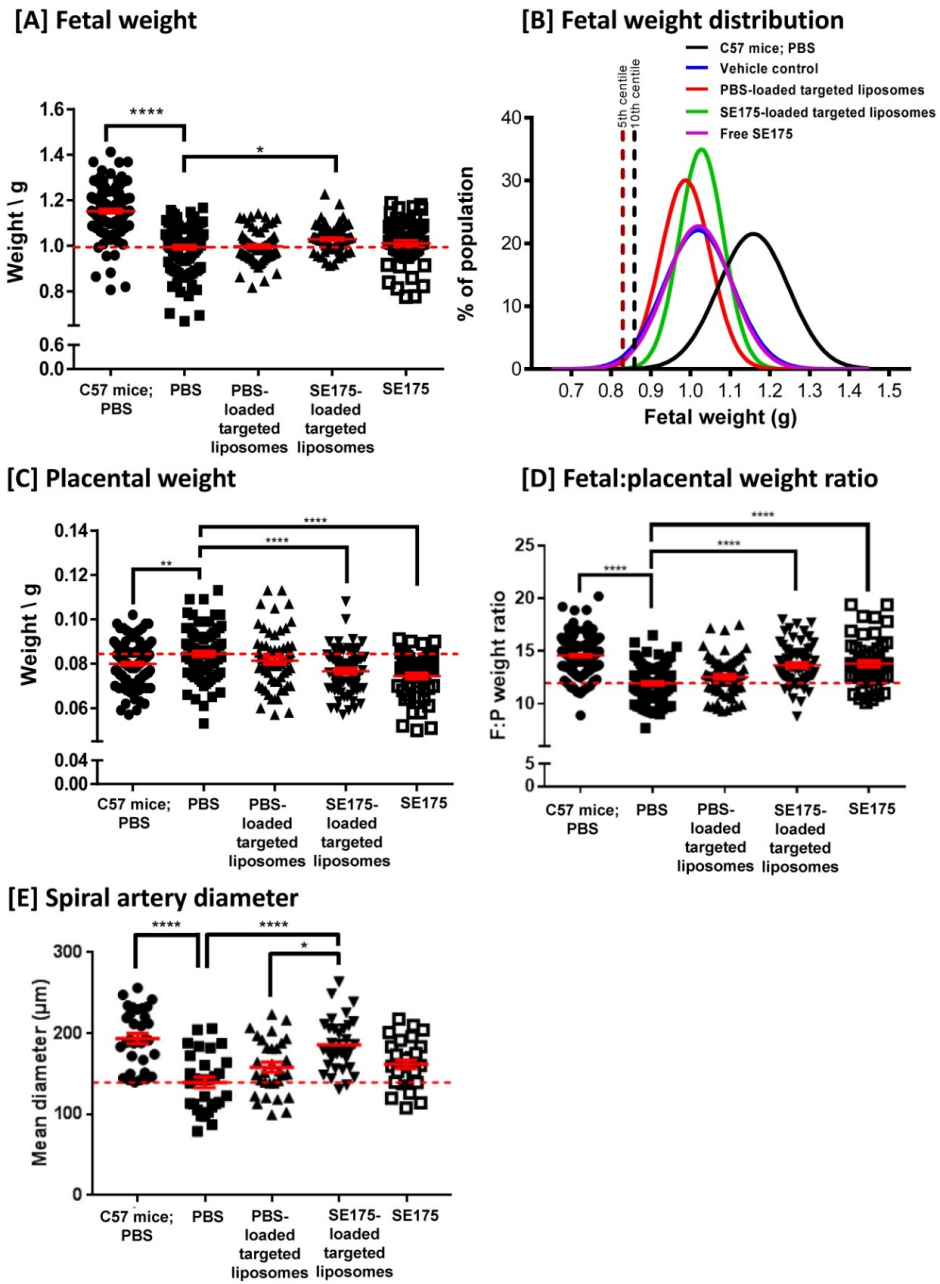
** Targeted delivery of SE175 increases fetal weight and placental efficiency in eNOS^-/-^ mice.** eNOS^-/-^ (N = dams, n = fetuses) were intravenously injected with 100 µL of PBS (N = 14, n = 113; closed black square), CNKGLRNK-decorated liposomes containing PBS (N = 9, n = 67; upward facing black triangle), CNKGLRNK-decorated liposomes containing SE175 (N = 10, n = 68; downward facing black triangle) or free SE175 (N = 8, n = 57; open black square). Data from PBS-treated C57BL/6J mice are shown for comparison (N = 17, n = 115; closed black circle).** [A]** Fetal weights, **[B]** curve fits to fetal weight distributions, **[C]** placental weights, **[D]** fetal:placental weight ratio (F:P), **[E]** spiral artery diameter (n = 3 placentas from N = 3 mice were assessed). Data points represent individual fetuses, placentas or arteries; mean ± SEM. Horizontal red dashed line represents vehicle control mean. Means were compared using one-way ANOVA with Dunnett's post hoc test. *P<0.05, **P<0.006, ****P<0.0001.

To determine whether SE175 could increase fetal growth by enhancing uteroplacental blood flow, pregnant wild-type C57BL/6J mice were intravenously injected with PBS, PBS-loaded CNKG-decorated liposomes, SE175-loaded CNKG-decorated liposomes or free SE175 on E11.5, E13.5, E15.5 and E17.5 of pregnancy. Whilst the treatments were well tolerated and did not significantly alter litter size or proportion of resorptions, targeted delivery of SE175 to the uteroplacental vasculature did not significantly alter fetal or placental weights, and administration of free SE175 modestly but significantly decreased fetal weight (Supplementary Figure 2). In contrast, when administered to eNOS**^-/-^** mice, a model of pre-pregnancy hypertension and FGR, targeted SE175 increased fetal weight (Figure 4A); no other treatment had a significant effect. Fetuses from eNOS^-/-^ mice had a mean weight 13 % lower than that of C57BL/6J mice; targeted delivery of SE175 increased mean fetal weight by 4 % compared to PBS-treated eNOS^-/-^ mice. After targeted SE175 treatment no fetuses fell below the 5^th^ or 10^th^ weight centiles, thresholds that are commonly used clinically to define FGR (Figure 4B). Importantly, fewer fetuses with the lowest weights were observed in the treatment group, whereas the number of fetuses of appropriate weight was not changed (Figure 4A). In addition, no treatment significantly altered litter size or the resorption rate (Supplementary Figure 3). This suggests that targeted delivery of SE175 increases the weight of the smallest fetuses, without causing detrimental overgrowth of the heavier fetuses.

eNOS^-/-^ placentas were on average 6 % heavier than those of healthy C57BL/6J; targeted delivery of SE175 significantly reduced placental weight by an average of 9 %, compared to PBS-treated eNOS^-/-^ mice (Figure 4C). Placental efficiency, described in terms of the fetal:placental weight ratio, was on average 18 % lower in PBS-treated eNOS^-/-^ mice than in PBS-treated C57BL/6J mice; targeted delivery of SE175 significantly increased F:P weight ratio in eNOS^-/-^ mice by an average of 15 % compared to PBS treatment (Figure 4D). Delivery of free SE175 also significantly decreased placental weight and enhanced fetal:placental weight ratio compared to controls, but this improvement in placental efficiency did not translate into increased fetal weights.

Maternal blood is supplied to the placenta via the decidual spiral arteries. Quantification of mean arterial diameter revealed that eNOS^-/-^ arteries were on average 28 % narrower than those of C57BL/6J; targeted delivery of SE175 significantly increased spiral artery diameter by an average of 33 % compared to PBS-treated eNOS^-/-^ mice, and 17 % compared to PBS liposome-treated eNOS^-/-^ mice (Figure 4E). Systemically administered SE175 had no significant effect on mean arterial diameter. All of the observed effects were independent of fetal sex.

### Targeted delivery of SE175 significantly reduces placental oxidative stress

**Figure 5:**
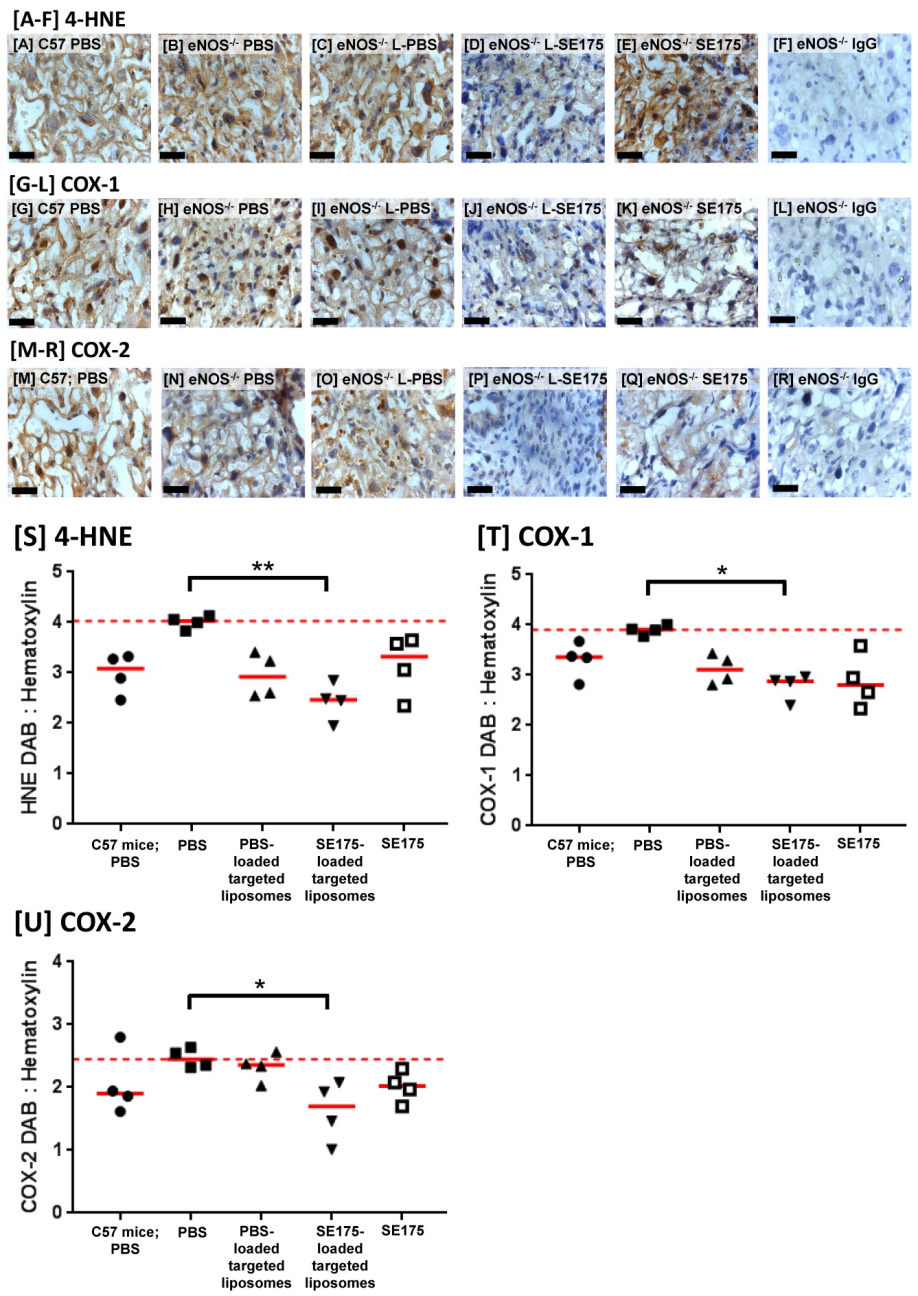
** Targeted delivery of SE175 reduces expression of the oxidative stress markers 4-HNE, COX-1 and COX-2 in eNOS^-/-^ mice** Immunostaining of the mouse placental labyrinth with antibodies to **[A - F]** 4-HNE; **[G - L]** COX-1; **[M - R]** COX-2. **[A, G, M]** PBS-treated C57BL/6J mice, **[B, H, N]** PBS-treated eNOS^-/-^ mice; **[C, I, O]** eNOS^-/-^ mice treated with CNKGLRNK-decorated liposomes containing PBS (L-PBS); **[D, J, P]** eNOS^-/-^ mice treated with CNKGLRNK-decorated liposomes containing SE175 (L-SE175); **[E, K, Q]** eNOS^-/-^ mice treated with free SE175; **[F, L, R]** eNOS^-/-^ mice, IgG control. Blue = hematoxylin; Brown = DAB; scale bars = 50 µm. n = 3 placentas from N = 4 mice were assessed; representative images are shown. **[S-U]** Quantitative assessment of DAB:Hematoxylin ratio of total area of **[S]** HNE; **[T]** COX-1; **[U]** COX-2. Mean ± SEM. Horizontal red dashed line represents vehicle control mean. Means were compared using a Kruskal Wallis test with Dunnett's post hoc test. *P<0.05; **P<0.01.

Placentas from treated eNOS^-/-^ mice were immunostained for the lipid peroxidation product 4-hydroxynonenal (4-HNE), as a marker of oxidative stress; PBS-treated C57BL/6J mice were also included for comparison. HNE immunoreactivity was observed throughout the decidua, junctional zone and labyrinth of vehicle treated eNOS^-/-^ mouse placentas. The area of immunopositive tissue was quantified and expressed as a ratio of the total tissue area; targeted delivery of SE175 significantly reduced the area of placental 4-HNE immunostaining, suggestive of a reduction in lipid peroxidation in eNOS^-/-^ mice (Figure 5A). No significant differences were observed in any other treatment groups. Placentas were also immunostained for COX-1 and COX-2; immunoreactivity was observed throughout the decidua, junctional zone and labyrinth of eNOS^-/-^ placentas. Targeted delivery of SE175 also significantly reduced the area of COX-1 and COX-2 immunostaining (Figure 5B, C), and no treatment significantly increased COX expression, lending support to the safety of treatment. The observed effects were independent of fetal sex.

### SE175 does not alter human placental growth or function *ex vivo*

**Figure 6:**
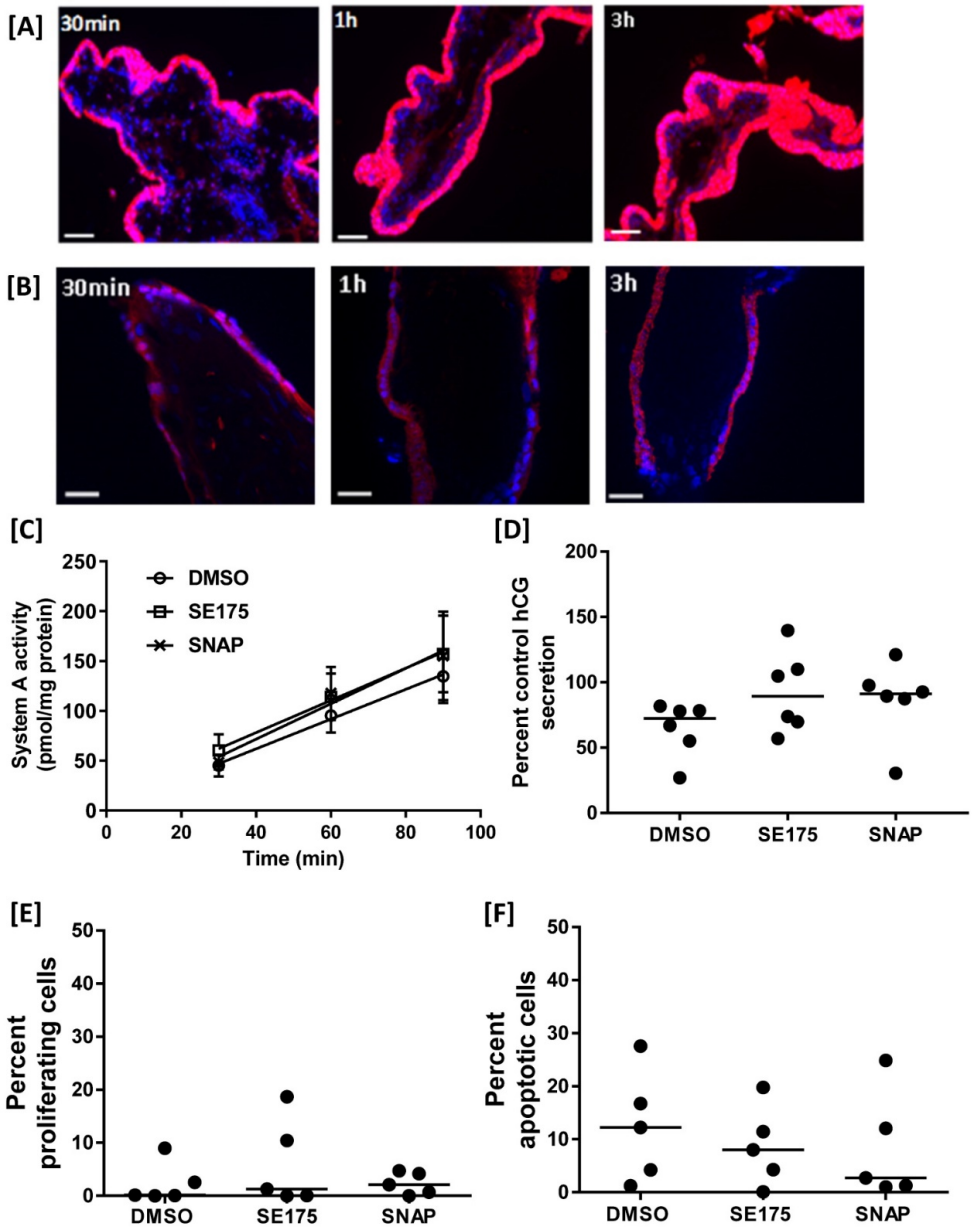
** SE175 does not alter human placental growth or function [A]** First trimester and **[B]** term human placental explants were incubated with Rh-CNKGLRNK (0.27 µmol/L) for up to 3 h. Peptide binding and uptake were assessed by fluorescence microscopy; (n = 3). Representative images are shown. Rh-labelled peptides, red; DAPI (nuclei), blue. ST = syncytium; VS = villous stroma; scale bar = 50 µm. **[C-F]** Term placental explants were treated with DMSO (vehicle control), SE175 or the nitric oxide donor SNAP for 24h.** [C]** Na^+^-dependent [^14^C]MeAIB uptake at 30, 60 and 90 min; mean ±SEM; n = 6. **[D]** Percent control hCG secretion as measured by ELISA; mean, n = 6. **[E]** Percent of Ki67-positive (in-cycle) cells; mean, n = 5.** [F]** Percent of M30-positive (apoptotic) cells; mean, n = 5.

To determine whether targeted delivery of SE175 would detrimentally affect human trophoblast function, Rh-CNKG and SE175 were incubated with human placental explants. Rh-CNKG exhibited cell-penetrating properties, binding to and accumulating within the outer syncytiotrophoblast (STB) layer of first trimester explants within 30 minutes (Figure 6A), and to a lesser extent in the STB of term placental explants (Figure 6B); minimal penetration of the peptide into the underlying villous stroma was observed in either tissue. These results suggested that SE175 would be delivered to the placental surface if administered via CNKG-loaded liposomes. To determine whether SE175 treatment altered any important parameters of placental growth or function, term explants were treated with SE175 for 24 h; the nitric oxide donor SNAP was included as a positive control. SE175 treatment did not significantly alter the rate of amino acid transport, as measured by Na^+^-dependent ^14^C meAIB uptake (Figure 6C), hCG secretion, a measure of trophoblast cell differentiation (Figure 6D), the basal rate of proliferation (Figure 6E) or apoptosis (Figure 6F).

## Discussion

Here we present evidence that the placental-specific homing peptide sequence CNKGLRNK acts as an efficient cell-penetrating peptide and can be conjugated to biocompatible liposomes. The resultant preparation selectively binds to the outer syncytiotrophoblast layer of human placenta *ex vivo,* and to the maternal uterine arteries and vasculature of the placental labyrinth in mice. We show that a novel vasodilator compound, the NO donor SE175, is able to induce vasodilation of mouse uterine arteries and human chorionic plate arteries *ex vivo*. We detected no adverse effect of SE175 on several parameters of human placental growth and function and peptide-decorated liposomes encapsulating SE175 are well tolerated by pregnant mice. Finally, targeted delivery of SE175 to pregnant eNOS^-/-^ mice, which exhibit dysregulated vascular function and FGR, enhances fetal growth such that the smallest fetuses are elevated above the tenth centile of weight, a commonly used clinical definition of growth restriction. Placental weight is decreased and placental efficiency is significantly enhanced. Furthermore, mean spiral artery diameter is significantly increased, and placental expression of the oxidative stress markers COX-1, COX-2 and HNE is significantly reduced, suggestive of enhanced uteroplacental perfusion.

FGR is a significant cause of stillbirth, neonatal morbidity and mortality 1,2, as well as developmental programming, which increases the risk of chronic diseases in adulthood 43. As management strategies are limited and there are currently no therapeutics licensed for use, it is of utmost importance to identify new approaches for the treatment of FGR *in utero*. A major barrier to drug development in obstetrics is the risk of side effects in mother or fetus. A strategy that has proven useful in limiting off-target side effects of chemotherapy has been the development of targeted drug delivery systems 10. We have previously demonstrated the potential of peptide-conjugated liposomes as a means of selectively delivering a drug payload to the placenta, resulting in enhanced fetal growth in the absence of systemic side effects. We showed that targeted placental delivery of insulin-like growth factor-II (IGF-II) was more effective than systemically administered free IGF-II in improving fetal growth in the P0 mouse model of FGR 16. That work used a previously identified tumour-homing sequence that exhibited high affinity for uteroplacental tissues. In the present study, we took a different approach, using phage screening to identify novel homing sequences that selectively target the maternofetal interface in mice. Multiple rounds of phage biopanning led to the identification of numerous sequences with specificity for the uteroplacental vasculature; of the individual sequences tested, NKGLRNK exhibited the high degree of specificity for this vascular bed, and was not observed in fetal tissues, so was selected for incorporation into our liposome formulation. Interestingly, when using these nanoparticles for SE175 delivery, we observed comparable results to King *et al.* (2016): targeted SE175 significantly increased mean fetal weight and appeared to be of most benefit to the smallest fetuses within each litter, yet the weight of the largest fetuses was not increased. This suggests that like targeted IGF-II 16, targeted SE175 acted preferentially on fetuses at most need of a growth stimulus, namely those in which growth restriction equated to a widely accepted clinical definition of FGR. We hypothesize that maternal and/or placental regulatory mechanisms exist to prevent overgrowth of healthy fetuses in response to external stimuli; thus, the beneficial effects of SE175 were limited to the smallest placentas and fetuses within the litter.

There is accumulating evidence in humans that impaired blood flow through the uteroplacental unit occurs in some cases of FGR 4, and that abnormal vascular anatomy 17,44 and impaired myometrial and chorionic plate vascular function contribute to the underlying pathophysiology 18-22. In parallel, there has been increasing interest in the use of vasodilators such as sildenafil citrate 26-28, carbon monoxide 45-47 and hydrogen sulphide 48-50 as putative therapeutics in mice and humans. However, these compounds are not without side effects; maternal sildenafil citrate administration impaired fetal aortic vascular function, reduced fetal glucose tolerance and caused elevated blood pressure in adulthood in mice 27,31, and caused fetal cerebral blood overflow in the pregnant rabbits 51. In mice, carbon monoxide exposure in pregnancy has been linked to increased resorptions 52, fetal skeletal variants 53, gross fetal malformations 52 and learning impairment in offspring 54. Hydrogen sulphide is a mucous membrane and respiratory tract irritant and an inhibitor of the cytochrome c oxidase system that can cause anoxia. Thus, we sought to examine the therapeutic potential of another compound, the nitric oxide donor SE175 55. SE175 has a short half-life *in vivo* and therefore a short diffusion distance once released. As it spontaneously liberates NO following cleavage by cell-surface esterases, development of tolerance is much less likely than with traditional nitrate-based therapies 56. Here we demonstrate that both free- and liposomally encapsulated SE175 causes vasorelaxation of pre-constricted mouse wild-type and eNOS^-/-^ uterine arteries and human chorionic plate arteries. Furthermore, SE175 did not detrimentally alter the basal rate of proliferation, apoptosis, differentiation or amino acid transport in human term placental explants, nor alter placental growth and function in mice, suggesting it may be a promising candidate for use in women. As NO has been implicated as a regulator of a number of important trophoblast functions including cell turnover, invasion and protection from apoptosis 57-59, this is a very positive outcome.

*In vivo*, targeted delivery of SE175 significantly decreased placental expression of the oxidative stress marker 4-HNE, an unsaturated hydroxyalkenal that is produced by lipid peroxidation in cells. Oxidative stress has previously been shown to induce upregulation of COX enzyme expression in the mouse placenta 37; in our study, expression of COX-1 and COX-2 was also reduced by SE175. These findings imply that either SE175 significantly enhanced local blood flow to reduce oxidative stress, or that it exhibits additional antioxidant properties. Given that liposomally-encapsulated SE175 also increased mean spiral artery diameter, this provides more evidence for improved uteroplacental perfusion following administration of SE175. Suboptimal placental function is characterised by increased placental oxidative stress 60 which in turn can lead to vascular dysfunction 61, so this observation highlights another potential benefit of SE175.

It has been hypothesised that the salicylate-like breakdown product of SE175 may act as an inhibitor of cyclooxygenase (COX) activity 55. As COX-1 expression is elevated in the placentas of women with pre-eclampsia 62, and COX inhibition improves fetal growth and attenuates late gestational hypertension in mice 63, we sought to examine whether SE175 exerts beneficial effects on human placental growth and function that are additional to those associated with the NO donor SNAP, which does not inhibit COX. There was no significant difference between the effects of SNAP and SE175 on trophoblast turnover, amino acid uptake or hCG secretion in human term placental explants; thus, either SE175 did not reach a concentration high enough to inhibit COX activity, or COX inhibition had no effect on the parameters tested.

Much of the previous work on targeted nanoparticles has focussed on delivery of chemotherapeutics to tumours; as such, the biocompatibility and safety of the nanocarriers has been considered of lesser importance, with any reduction in systemic side effects being regarded as advantageous. Moreover, as the primary aim is to eliminate the tumour cells, any tissue-specific cytotoxicity is viewed as an additional positive outcome. The evaluation of nanoparticle effects on the placenta and its vasculature requires a more rigorous approach to the detection of toxicity. Studies of nanoparticle biodistribution and fetoplacental toxicity in pregnancy have determined the effects of size, surface charge and composition on outcome (reviewed by 64). The emerging picture indicates that the majority of the nanoparticles assessed, including quantum dots, silica, gold and iron oxide nanoparticles, exhibit significant placental transfer, fetal accumulation and cytotoxicity. Thus, liposomes represent an attractive option for nanoparticle-mediated placental drug targeting. The peptide-decorated liposomes used in this study did not cross the placenta, did not accumulate in fetal tissues and repeated administration did not cause any apparent fetal toxicity or physical deformities. In addition, treated dams did not display any signs of distress, and gross morphological analysis of maternal organs indicated no apparent abnormalities. Litter size and the number of resorptions were similar between treatment groups. However, since sequential measurements cannot be taken from the same animal with the methodologies used in this study, additional work is required to further understand nanoparticle absorption, distribution, metabolism and excretion, along with a detailed study of neonatal outcome.

Development of novel therapeutics for use in pregnancy is an area that has historically proved difficult to translate from the bench to the clinic. However, there is now a precedent for this: sildenfil citrate 65 and melatonin 66 are undergoing clinical trials for treatment of fetal growth restriction 67, and a novel vascular endothelial growth factor (VEGF) adenoviral gene therapy, which significantly increases uterine blood flow and fetal growth in guinea pigs and sheep, is being tested in humans 68-71. Thus, our novel system for selectively targeting the uteroplacental vasculature and identification of SE175 as a safe and effective uteroplacental vasodilator holds much promise for the future.

## Supplementary Material

Supplementary figures.

## Figures and Tables

**Table 1:** Primary antibodies

Antigen	Host Species	Target Species	Supplier	Dilution
COX-1 aa 274 - 288 #160109	Rabbit	Mouse	Cayman Chemicals, UK	1:250
COX-2 aa 584 - 598 #160126	Rabbit	Mouse	Cayman Chemicals, UK	1:1000
HNE #HNE11-S	Rabbit	Mouse	Alpha Diagnostics, UK	1:500
Ki67 (clone MIB-1)	Mouse	Human	DakoCytomation A/S, Denmark	1:250
M30 CytoDEATH	Mouse	Human	Roche Diagnostics GmbH, UK	1:100
IgG #I8140	Rabbit	Mouse	Sigma Aldrich, UK	Matched
IgG #I8765	Mouse	Human	Sigma Aldrich, UK	Matched

## Abbreviations

4-HNE: 4-hydroxynonenal
CNKG: CNKGLRNK
COX-1: cyclooxygenase-1
COX-2: cyclooxygenase-2
DAB: diaminobenzidine
DPSC: 1,2-distearoyl-*sn*-glycero-3-phosphocholine
DSPE-PEG: 1,2-distearoyl-*sn*-glycero-3-phosphoethanolamine-N-[amino(polyethylene glycol)]
E: embryonic day
eNOS^-/^: endothelial nitric oxide synthase knockout
FAM: 5(6)-carboxyfluorescein
FGR: fetal growth restriction
IGF-II: insulin-like growth factor-II
JZ: junctional zone
Lab: labyrinth
NKG: NKGLRNK
NO: nitric oxide
rh: rhodamine
SA: spiral artery
SE175: 2-[[4-[(nitrooxy)methyl]benzoyl]thio]-benzoic acid methyl ester
SNAP: S-nitroso-N-acetylpenicillamine
STB: syncytiotrophoblast
VEGF: vascular endothelial growth factor.
