# Supplementary material for: Selective Targeting of a Novel Vasodilator to the Uterine Vasculature to Treat Impaired Uteroplacental Perfusion in Pregnancy
Source: Theranostics. 2017 Aug 29;7(15):3715–31. doi: 10.7150/thno.19678 (PMC5667343; doi:10.7150/thno.19678)
Supplement: Supplementary file 1 — Supplementary figures. [file thnov07p3715s1.pdf]

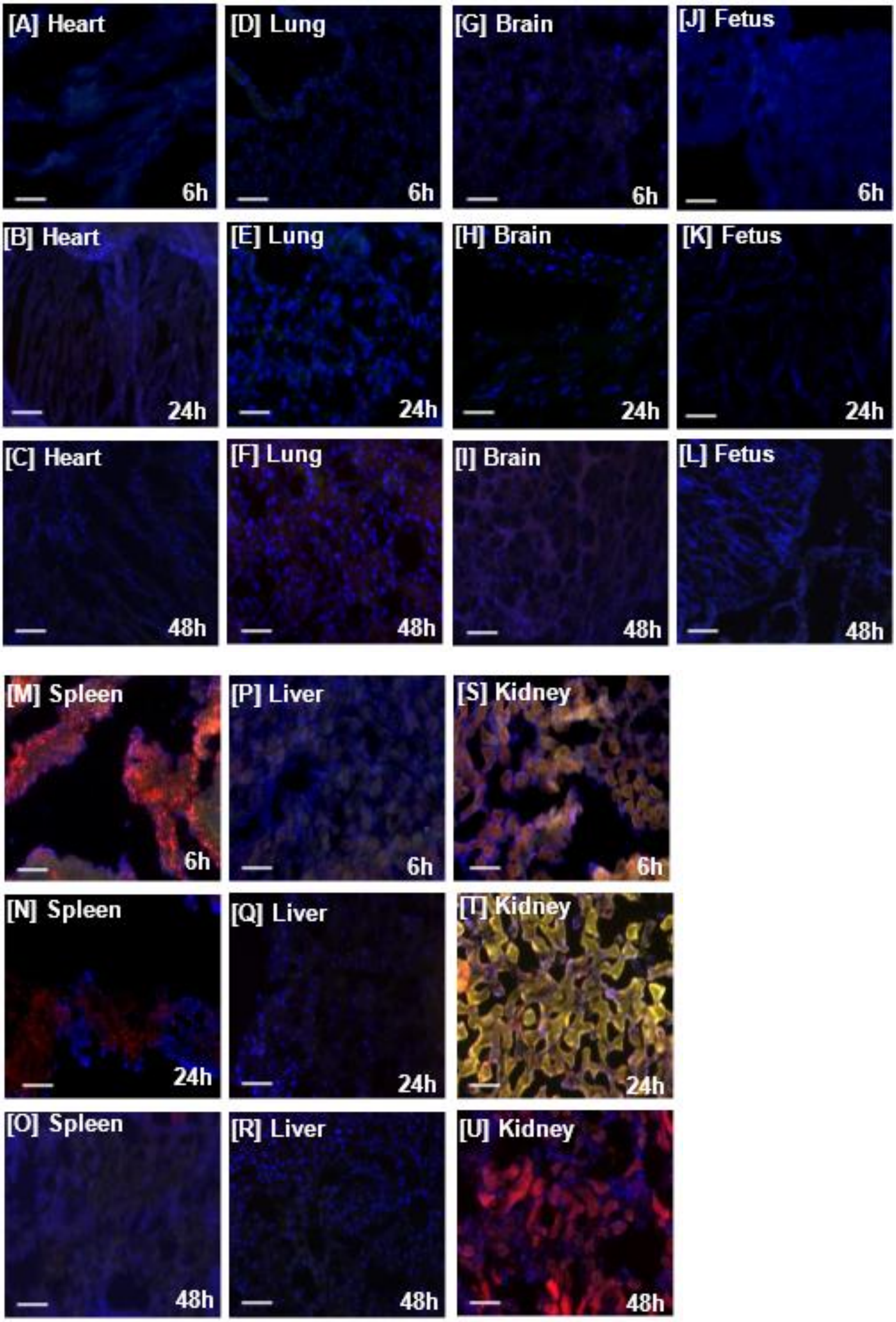
[A] Heart
6h
[B] Heart
24h
[C] Heart
48h
[D] Lung
6h
[E] Lung
24h
[F] Lung
48h
[G] Brain
6h
[H] Brain
24h
[I] Brain
48h
[J] Fetus
6h
[K] Fetus
24h
[L] Fetus
48h
[M] Spleen
6h
[N] Spleen
24h
[O] Spleen
48h
[P] Liver
6h
[Q] Liver
24h
[R] Liver
48h
[S] Kidney
6h
[T] Kidney
24h
[U] Kidney
48h

Supplementary Figure 1: Identification of the placental-homing peptide NKGLRNK

[A-U] Maternal organs and fetuses from pregnant C57BL/6J mice collected at E18.5, following tail vein injection of Rh-CNKGLRNK (red)-decorated liposomes composed of NBD-labelled lipids (green), 6, 24 or 48h prior to tissue harvest. Cardiac perfusion was performed to remove unbound peptide; organs were removed and assessed by fluorescence microscopy. Blue, DAPI (nuclei). Scale bar = 50 µm. Tissues from n = 3 mice per treatment group were examined. Representative images are shown.

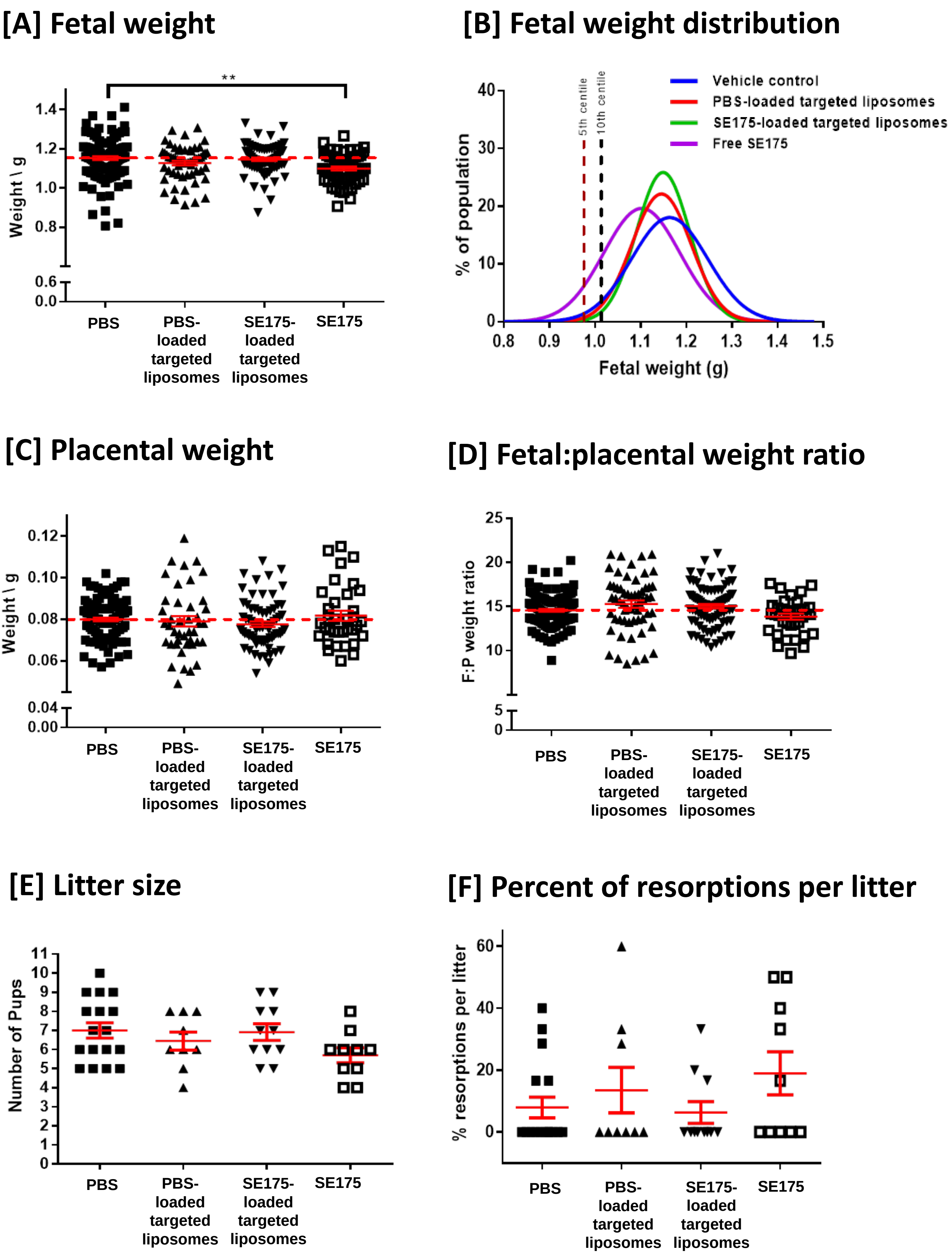
[A] Fetal weight
**
Weight \ g
1.4
1.2
1.0
0.8
0.6
0.0
PBS
PBS-loaded targeted liposomes
SE175-loaded targeted liposomes
SE175
[B] Fetal weight distribution
5th centile
10th centile
Vehicle control
PBS-loaded targeted liposomes
SE175-loaded targeted liposomes
Free SE175
% of population
40
30
20
10
0
0.8
0.9
1.0
1.1
1.2
1.3
1.4
1.5
Fetal weight (g)
[C] Placental weight
Weight \ g
0.12
0.10
0.08
0.06
0.04
0.00
PBS
PBS-loaded targeted liposomes
SE175-loaded targeted liposomes
SE175
[D] Fetal:placental weight ratio
F:P weight ratio
25
20
15
10
5
0
PBS
PBS-loaded targeted liposomes
SE175-loaded targeted liposomes
SE175
[E] Litter size
Number of Pups
11
10
9
8
7
6
5
4
3
2
1
0
PBS
PBS-loaded targeted liposomes
SE175-loaded targeted liposomes
SE175
[F] Percent of resorptions per litter
% resorptions per litter
60
40
20
0
PBS
PBS-loaded targeted liposomes
SE175-loaded targeted liposomes
SE175

Supplementary Figure 2: Targeted delivery of SE175 has no effect on fetal or placental weight in C57BL/6J mice

C57BL/6J mice (N = dams, n = fetuses) were intravenously injected with 100 μL of PBS (N = 17, n = 115; closed black square), CNKGLRNK-decorated liposomes containing PBS (N = 9, n = 58; upward facing black triangle), CNKGLRNK-decorated liposomes containing SE175 (N = 11, n = 72; downward facing black triangle) or free SE175 (N = 10, n = 56; open black square). Horizontal red dashed line represents vehicle control mean. **[A]** Fetal weights, **[B]** curve fits to fetal weight distributions, **[C]** placental weights, **[D]** fetal:placental weight ratio (F:P), **[E]** litter size, **[F]** percent of resorptions per litter. Data points represent individual fetuses or placentas; mean ± SEM. Means were compared using one-way ANOVA with Dunnett's *post hoc* test. **P<0.001.

[A] Litter size

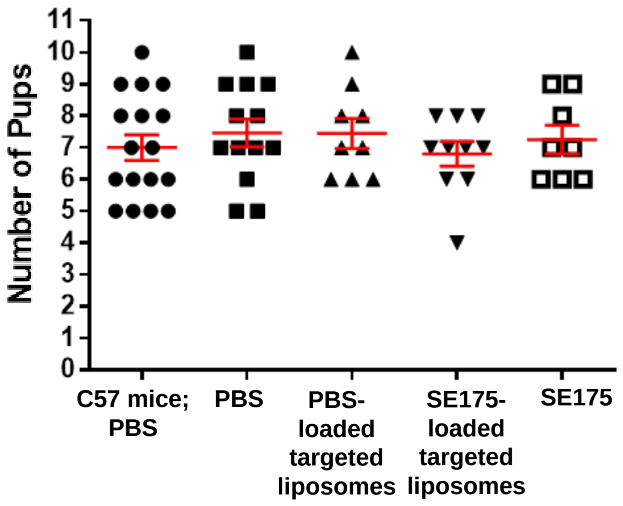

[B] Percent of resorptions per litter

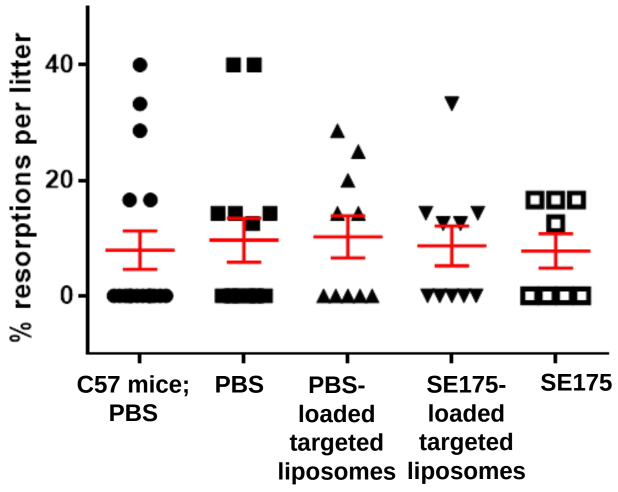

Supplementary Figure 3: Targeted delivery of SE175 does not alter litter size or resorption rate in eNOS$^{-/-}$ mice

eNOS$^{-/-}$ (N = dams, n = fetuses) were intravenously injected with 100 µL of PBS (N = 14, n = 113; closed black square), CNKGLRNK-decorated liposomes containing PBS (N = 9, n = 67; upward facing black triangle), CNKGLRNK-decorated liposomes containing SE175 (N = 10, n = 68; downward facing black triangle) or free SE175 (N = 8, n = 57; open black square). Data from PBS-treated C57BL/6J mice are shown for comparison (N = 17, n = 115; closed black circle). **[A]** Litter size **[B]** Percent of resorptions per litter. Mean ± SEM.
